# Identification of Molecular Fluorophore as a Component of Carbon Dots able to Induce Gelation in a Fluorescent Multivalent-Metal-Ion-Free Alginate Hydrogel

**DOI:** 10.1038/s41598-019-51512-2

**Published:** 2019-10-21

**Authors:** Peter Kasak, Martin Danko, Sifani Zavahir, Miroslav Mrlik, Yuan Xiong, Ammar Bin Yousaf, Wing-Fu Lai, Igor Krupa, Jan Tkac, Andrey L. Rogach

**Affiliations:** 1 Center for Advanced Materials, Qatar University, P.O. Box 2713, Doha, Qatar; 2 Polymer Institute, Slovak Academy of Sciences, Dúbravská cesta 9, 845 41 Bratislava, Slovak Republic; 3 Centre of Polymer Systems, University Institute, Tomas Bata University in Zlín, Trida T. Bati 5678, 760 01 Zlín, Czech Republic; 4 Department of Materials Science and Engineering, and Center for Functional Photonics (CFP), City University of Hong Kong, 83 Tat Chee Avenue, Kowloon, SAR Hong Kong; 5 School of Pharmaceutical Sciences, Health Science Center, Shenzhen University, Shenzhen, China; 6 Department of Glycobiotechnology, Institute of Chemistry, Slovak Academy of Sciences, Dúbravská cesta 9, 845 38 Bratislava, Slovak Republic

**Keywords:** Gels and hydrogels, Self-assembly

## Abstract

We introduce a simple approach to fabricate fluorescent multivalent metal ion-free alginate hydrogels, which can be produced using carbon dots accessible from natural sources (citric acid and L-cysteine). Molecular fluorophore 5-oxo-2,3-dihydro-5H-[1,3]-thiazolo[3,2-a] pyridine-3,7-dicarboxylic acid (TPDCA), which is formed during the synthesis of carbon dots, is identified as a key segment for the crosslinking of hydrogels. The crosslinking happens through dynamic complexation of carboxylic acid groups of TPDCA and alginate cages along with sodium ions. The TPDCA derived hydrogels are investigated regarding to their thermal, rheological and optical properties, and found to exhibit characteristic fluorescence of this aggregated molecular fluorophore. Moreover, gradient hydrogels with tunable mechanical and optical properties and controlled release are obtained upon immersion of the hydrogel reactors in solutions of divalent metal ions (Ca^2+^, Cu^2+^, and Ni^2+^) with a higher affinity to alginate.

## Introduction

Naturally occurring polymers have received great attention as useful materials in particular for food science applications. This is partly due to their biocompatibility, biodegradability, non-immunogenicity, affordable cost, and last but not least an easy gelation ability^1–5^. Alginate, as an accessible source of polyanionic polysaccharides from brown algae^6^, is one of the industrially useful biopolymers^7,8^. Alginates consist of linearly linked homopolymeric block segments of (1,4)-linked β-D-mannuronate (M) and C-5 epimer α-L-guluronate (G), with various ratios of G/M segments based on the source^9^. The most commonly used form of alginate is its gel crosslinked with the aid of multivalent metal ions, such as Ca^2+^ or Ba^2+^ cations^10,11^. These cations interact electrostatically with carboxylate ions from the G segments of alginate, and create crosslinking point segments (“egg boxes”) resulting in stable 3D polymeric network^12,13^. However, several applications of the gels benefit from the absence of any multivalent metal ions in the hydrogel structure. Various approaches to crosslink alginates without employment of divalent metal ions have been suggested^14^, such as a modified dopamine structure and its oxidative crosslinking^15^, glutaraldehyde crosslinking^16^, photo-crosslinking with anchored methacrylate moieties^17–19^, and amidation of alginate^20^ or boronic acid modified alginate^21,22^. However, all these approaches require chemical modification of alginate polymer chains.

Recently, Diaz Diaz group introduced a multivalent metal-ion-free hydrogel formation from alginate triggered by dimethyl sulfoxide or oxalic acid^23^. It was claimed that interactions between alginate chains and dimethyl sulfoxide molecules are responsible for the 3D polymer network formation, while in the case of oxalic acid, the gel was formed from simultaneous association among oxalate, alginate and Na^+^ counterions. The resulting gels have rather limited application prospects due to harmfulness and safety issues of both dimethyl sulfoxide and oxalic acid components. The search for other, more environmentally friendly multivalent metal-ions-free gelating agents for the alginates is thus ongoing. In particular, carbon dots (CDs) have triggered a lot of interest from the research community as a promising material for optoelectronic, biomedical and catalytic applications, which are easy to synthesize from the affordable, nontoxic precursors^24^. One of the most popular and easily up-scalable methods for synthesis of CDs utilizes the cheap, natural and sustainable precursor, citric acid (CA), as the carbon source^25,26^, often combined with α,β-diamines, α-aminoacids or α,β-heteroatom amines as the nitrogen doping source^27,28^. CDs can be easily entrapped within different matrices for further processing, such as silica based immobilization^29,30^, CD-based ionogel composites^31,32^ or polyurethane based incorporation^33^. Interestingly, carbon nanomaterials such as carbon nanotubes were recently demonstrated to show positive effects on gel formation^34^. Steed *et al*. utilized CDs as nucleation centers and cross-linking nodes, where hydrophobic and π-π stacking interactions enabled formation of self-assembled fibrillar networks^35^. More recently, Rizzo *et al*. applied nitrogen-doped CDs as gelating agents in ionic liquid solutions, which enabled fabrication of the gels with good thermal stability, mechanical strength, and antioxidant activity^32^.

We have recently reported that under the relatively mild synthetic conditions, molecular fluorophores are formed alongside inorganic CDs^36–39^. These studies followed pioneering work of Yang’s group, who identified the fluorescent citrazinic acid derivative 5-oxo-1,2,3,5-tetrahydroimidazo [1,2-α] pyridine-7-carboxylic acid (denoted as IPCA) as the major luminescent component formed during the hydrothermal synthesis of CDs from CA and ethylenediamine^40^. This and another brightly luminescent molecular fluorophore of a similar structure, namely as 5-oxo-2,3-dihydro-5H-[1,3]-thiazolo[3,2-a]pyridine-3,7-dicarboxylic acid (TPDCA)^41^ have been reported as components of fluorescence biodegradable polymers^42,43^, and other kinds of soft composite materials^44–47^.

In this work, we screened contributions of the components of CDs synthesized from the reaction between CA and L-cysteine, such as the carbon core and the molecular fluorophores in terms of their potential ability to form gels with alginate. After identification of the molecular fluorophore TPDCA as a key component to initiate gelation, we have conducted thorough rheological studies of TPDCA derived hydrogels, revealing their strong elasticity, thixotropic behavior and fast recoverability. Subsequent optical studies showed that the emission properties of luminescent hydrogels were determined by the aggregation state of the TPDCA. Additionally, gradient hydrogels with controlled release capacity have been obtained by immersion of TPDCA derived hydrogels into solutions of divalent metal ions (Ca^2+^, Ni^2+^ and Cu^2+^).

## Results and Discussion

### Screening of the gelating ability of the components of CDs

**Figure 1:**
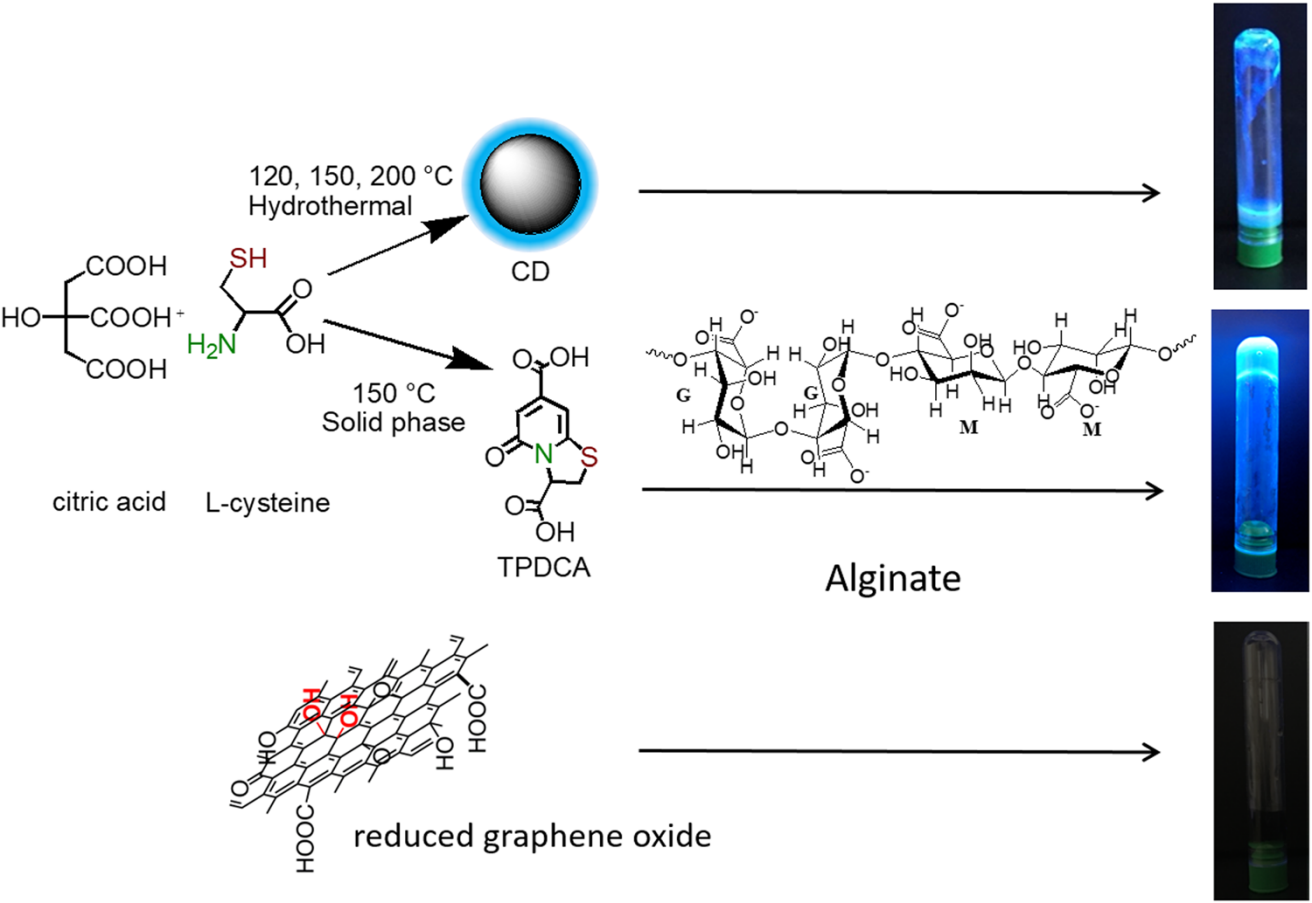
Schematic presentation of the synthetic pathways towards CDs, molecular fluorophore TPDCA and reduced graphene oxide, which were combined with alginate to eventually form fluorescent hydrogels. Photographs on the right, taken under excitation with a UV lamp, show appearance of the respective samples.

We have performed screening tests to find out which segments of CDs (carbon cores, modelled by GO and rGO as in the previous study^48^, molecular fluorophores, or CDs as a whole) contribute to gelation. CDs employed here were synthesized using a well-established procedure^49^, from the naturally occurring precursors citric acid (CA) and L-cysteine. CDs produced under different temperatures of the hydrothermal treatment, namely 120 °C, 150 °C and 200 °C, had the average lateral size of 2.3 ± 1 nm, 2.5 ± 1 nm and 3.0 ± 1 nm, respectively, as determined by AFM. As extensively reported in literature^49,50^, these CDs possess multiple nitrogen- and oxygen-containing functional groups at the surface, in particular carboxylic groups. The synthesized CDs were purified by dialysis and applied for the screening tests. To perform the comparison with two other components, namely purely carbon cores^48^ and the molecular fluorophore^41^, GO and rGO as the models of functionalized carbon-based aromatic domains, and an isolated dye molecule 5-oxo-2,3-dihydro-5H-[1,3]-thiazolo[3,2-a]pyridine-3,7-dicarboxylic acid (TPDCA) were also tested as potential gelation reagents (Fig. 1). All these screened materials were applied in a relative amount of 3.3 wt% for SA gel formation. The gelation ability and stability of the gels were tested after 16 h of gelation by stainless steel ball and inversion tests, and the related data are summarized in Table 1.

**Table 1:** Selected screening tests for the gelation ability of sodium alginate (SA) in the presence of different gelating reagents (GR).

GR	GR prepared by^a^	Conc. SA^b^	Appearance^c^	T_G_^d^	GR	GR prepared by^a^	Conc. SA^b^	Appearance^c^	T_G_^d^
CDs	HT 120 °C	1.8	VL	SM	TPDCA	S 150 °C	1.8	VL	SM
	HT 120 °C	3.6	VL	SM		S 150 °C	3.6	G	50
	HT 120 °C	5.4	VL	SM		S 150 °C	5.4	G	46
	HT 120 °C	7.2	G	30		S 150 °C	7.2	OG	45
	HT 150 °C	1.8	VL	SM	GO	NA	1.8	D	
	HT 150 °C	3.6	VL	SM		NA	3.6	D	
	HT 150 °C	5.4	VL	SM		NA	5.4	D	
	HT 150 °C	7.2	G	28		NA	7.2	D	
	HT 200 °C	1.8	VL	SM	rGO	AA, 90 °C	1.8	D	
	HT 200 °C	3.6	VL			AA, 90 °C	3.6	D	
	HT 200 °C	5.4	VL			AA, 90 °C	5.4	D	
	HT 200 °C	7.2	VL			AA, 90 °C	7.2	D	

^a^HT – hydrothermal reaction, S – solid state reaction, AA – ascorbic acid, NA – not applicable.

^b^Concentration of GR was constant at 3.3 wt% in all gels.

^c^Appearance: VL – viscous liquid, G – gel, OG – opaque gel, D – dispersion.

^d^T_G_ was determined by stainless steel ball technique, and is given in °C; SM – soft material that does not support the steel ball.

CDs synthesized at different temperatures showed different gel forming ability. It was weak but available for CDs synthesized at 120 °C (Table 1 and Fig. S3a); soft material was formed with 1.8, 3.6 and 5.4% of SA, while the sample with 7.2% SA was able to form a stable hydrogel that sustained steel ball test with a relative weak transition at 30 °C. Even weaker gelation ability was observed for CDs synthesized at 150 °C; a stable hydrogel in this case could be formed only with 7.2% of SA, but the transition temperature was even lower, namely 28 °C (Table 1). No gels could be formed for CDs synthesized at 200 °C (Table 1). Graphene oxide and reduced graphene oxide-based samples did not form any gels, either; just dispersion with sedimentation was obtained, and solution did not show any fluorescence (Table 1, Fig. 1). Such a clear tendency on the decreasing gelation ability of CDs synthesized at increasing temperature can be explained by the influence of the latter on the formation of molecular fluorophore species within hydrothermal synthesis of CA-based CDs^38,51^. For the hydrothermal reactions used for synthesis of CDs and conducted at rather low temperatures, the molecular fluorophore is often a predominant component and a source of the fluorescence^36–39^. The higher degree of carbonization of CA and amine precursors, which results in the predominant formation of graphitic carbon cores similar to GO or rGO^48^, does not lead to the gelation ability of the resulting samples.

Our findings indicate that it is a molecular fluorophore as component of the nominally CD samples which can trigger the gelation process of SA, accompanied at the same time with an intense blue fluorescence of the resulting hydrogels (Fig. 1). Isolated molecular fluorophore TPDCA has indeed shown the strongest gelation ability, when applied for the SA gel formation (Table 1). There was no formation of bubbles observed during gelation, meaning that no exothermic reactions occurred. In fact, gelation is driven by a combination of attractive electrostatic forces and entropically-favorable molecular rearrangements^52,53^. Because of this, a decrease in the level of isolated molecular fluorophore TPDCA might decrease the entropic driving force for gel formation, thereby inhibiting the formation of the hydrogel^54,55^. A similar explanation can account for the influence of the TPDCA concentration on the gelation process as demonstrated in Table S1, and Fig. S3a. While the sample SA-1.8-L-3.3 was not able to form a gel (Fig. S3a upper left), the gelation was observed for the samples SA-3.6-L-3.3, SA-5.4-L-3.3 and SA-7.2-L-3.3, respectively. Thus, all the following experiments were performed with 3.3 wt% of TPDCA (33 mg in 1 ml) and variable SA content.

### TPDCA derived hydrogels

The hydrogel produced with the aid of TPDCA was injectable, as depicted in Fig. S3b. Such thixotropic behavior was further confirmed by the low and high strain change loop rheological experiments (vide infra), as described in detail below. In order to test the stability of the gel, it was mechanically agitated at ambient temperature; as demonstrated in Fig. S3c, the gel was transferable after agitation but could be restored with a comparable mechanical stability after 12 h while kept untouched.

**Figure 2:**
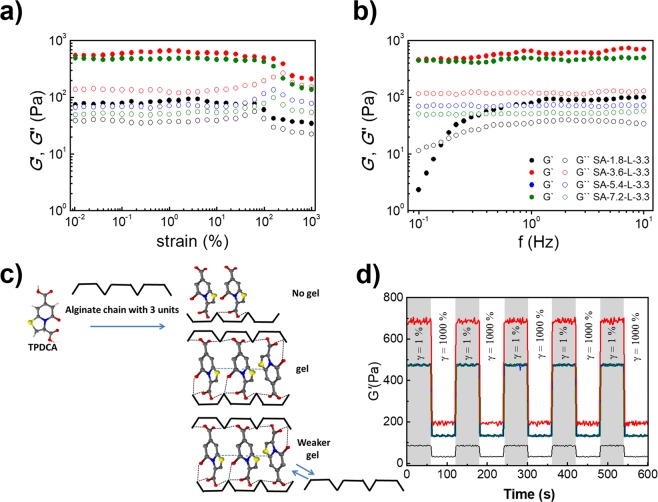
Strain (**a**) and frequency (**b**) dependence of the storage modulus, G′ (solid symbols) and viscous modulus, G′′ (open symbols) of the TPDCA derived hydrogels SA-1.8-L-3.3 (black), SA-3.6-L-3.3 (red), SA-5.4-L-3.3 (blue) and SA-7.2-L-3.3 (green). (**c**) Schematic presentation of the dynamic interaction with SA polymeric chain with a low amount of SA (upper), optimal molar ratio of TPDCA and SA (middle) and an excess of SA (bottom). (**d**) Self-healing and recovery tests for the TPDCA derived hydrogels SA-1.8-L-3.3 (black line), SA-3.6-L-3.3 (red line), SA-5.4-L-3.3 (blue) and SA-7.2-L-3.3 (green) at 25 °C at low (γ = 1%, grey region) and high strain (γ = 1000%, white region).

As can be seen in Fig. 2a, the sample SA-1.8-L-3.3 shows the lowest performance from the storage moduli point of view, namely 37 Pa in the linear viscoelastic region, and becomes significantly deformed over strain deformation of 100%. The increase of amount of SA to 3.6 wt% in the sample SA-3.6-L-3.3 brings an enormous increase in the storage moduli to 651 Pa which is almost 20 times higher than that of SA-1.8-L-3.3. The deformation of the SA-3.6-L-3.3 hydrogel is shifted to a higher value of strain, namely 400%. The further increase of the SA amount to 6 and 8 wt% does not bring any significant changes; samples SA-5.4-L-3.5 and SA-7.2-L-3.5 show storage moduli slightly lower than SA-3.6-L-3.3. However, the loss moduli decrease, indicating that cross-links within these gels become stronger, since the deformation is shifted to lower values of strain (200% and 180% for SA-5.4-L-3.3 and SA-7.2-L-3.3, respectively). Moreover, as can be seen from Fig. 2, even at 1000% strain all the samples SA-1.8-L-3.3, SA-3.6-L-3.3, SA-5.4-L-3.3 and SA-7.2-L-3.3 are still able to overcome deformation without any breakage, and no crossover point (G′ = G″) is observed. These observations are attributed to the phenomenon that the gelation process is driven partly by the entanglement of polymer chains and molecular interactions. When the amount of SA increases, the extents of polymer entanglement and molecular interactions increase too, leading to the formation of a gel that exhibits higher mechanical strength. Furthermore, G′ in all cases is higher that G″, and no flow (irreversible deformation) with a fluid-like behaviour takes place. This indicates the formation of a dynamic network in the hydrogels, where crosslinking points dynamically exchange to each other, thus ensuring gel-like behaviour even at high applied strains.

Frequency sweeps provide further information on how the TPDCA-based hydrogels are developed (Fig. 2b). In the case of SA-1.8-L-3.3, the cross-linking is rather low, as confirmed by the observed frequency dependence of both moduli. However, for SA-3.6-L-3.3, the well-developed network is already formed, as confirmed by the fact that both moduli are nearly frequency independent. Further increase of the amount of SA provides even less dependence on the frequency, as loss moduli decrease to 51 Pa and 71 Pa for SA-7.2-L-3.3 and SA-5.4-L-3.3, respectively. This indicates the increase of the strength of the prepared hydrogels for SA-3.6-L-3.3, most probably because TPDCA becomes cross-linked with the alginate structure at some optimal molar ratios, corresponding to ~1.5/1.8 for this particular sample. Carbohydrate monomeric units from SA interacting with TPDCA molecules are able to form crosslinking points in a balanced attraction between the alginate carbohydrate units, TPDCA molecules and Na^+^ ions (as shown schematically in Fig. 2c), similarly as was previously reported for oxalic acid interacting with alginate^23^. Consequently, a further excess of the SA amount weakens that stabile network and lowers the stiffness, and results in the possible irreversible deformation of hydrogels, which is visible from the decreasing G″ for the SA-5.4-L-3.3 and SA-7.2-L-3.3 samples.

Self-healing and recovery tests showed that all the TPDCA derived hydrogels provide an excellent recoverable performance. During the tests, the samples were subjected to alternated low strain (1%) and high strain (1000%) deformation, and the change of G′ over time was followed to document the recoverability (Fig. 2d). SA-1.8-L-3.3 was relatively weak in terms of the stiffness, but showed an excellent respond after the deformation of 1000% of strain. It is noteworthy that samples SA-5.4-L-3.3 and SA-7.2-L-3.3 were very similar, and overlapped in this measurement condition. Gels SA-3.6-L-3.3, SA-5.4-L-3.3 and SA-7.2-L-3.3 showed the values of storage moduli very close to dimethyl sulfoxide gelated alginate^23^, which were lower compared to the conventional divalent metal ions crosslinked alginate^56^, but higher than electrostatic interactions in the cellulose sulphate based gels^57^. Their significantly enhanced self-healing and recovery characteristics indicate strong affinity to reassembly, which is demonstrated by the fast and 100% recoverability and high strain loop in 5 cycles. This points out on the formation of a dynamical network in the resulting hydrogel. Taken all together, rheological characteristics of the TPDCA derived alginate-based hydrogels demonstrate tuneable viscoelastic characteristics, as well as excellent self-healing capability.

**Figure 3:**
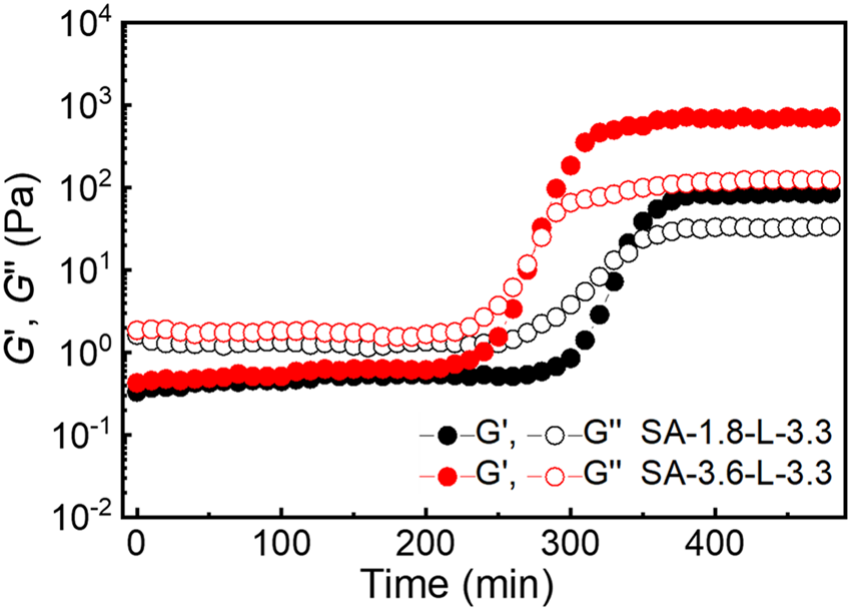
Rheological study of kinetics of gel formation for SA-1.8-L-3.3 (black dots) and SA-3.6-L-3.3 (red dots) at 22 °C (G′ values are given by full dots, and G″ - by empty dots).

From the rheological measurements performed over a period of time (Fig. 3), the formation of gels requires an induction period as a first, slower step for preorganization, before the second step of an entire gelation begins. Within the induction period, only a slight increase in G′ is observed over the first 200 min. It is assumed, that the origin of the cross-linking lies in a rather weak interaction between the alginate polymer chains and the cross-linker itself. The cross-linker possesses two functionalities, and it is proposed that its preorganization to properly complex SA and TPDCA requires some time as well as continuous oscillation (energy added to the system) to develop an ordered cross-linked structure. Similar observations are made in some previous study on a complex of DMSO and SA^23^. The crossover point G′ = G″ can be reached faster at a higher SA concentration, and the further increase of G′ over G″ leads to a plateau without any further change, from which the duration of the gel formation can be derived. For the sample S-1.8-L-3.3, the complete gelation was accomplished in 336 min, while for SA-3.6-L-3.3 – in 280 min (Fig. 3). For the samples S-5.4-L-3.3 and S-7.2-L-3.3, the gelation time was very similar, namely 275 and 274 min, respectively.

**Figure 4:**
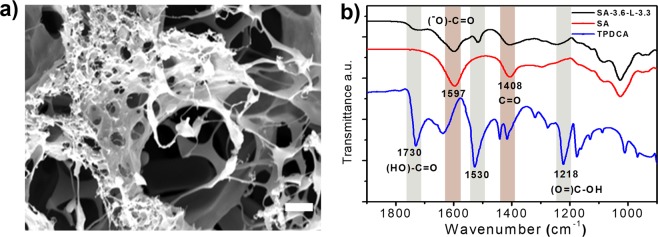
(**a**) SEM image of the freeze dried SA-3.6-L-3.3 xerogel. Scale bar is 2 µm. (**b**) FTIR spectra of the SA-3.6-L-3.3-based xerogel, SA and TPDCA.

The morphology of xerogels obtained from the TPDCA derived hydrogels by freeze drying was evaluated by SEM (Figs 4a and SI3). SEM images revealed formation of a dense fibrous network structure composed of the entangled fibers with widths ranging from 20 to 50 nm. Freeze dried xerogels exhibited pore sizes ranging from 50 μm for SA-1.8-L-3.3 down to app. 10 μm for SA-3.6-L-3.3, SA-5.4-L-3.3 and SA-7.2-L-3.3 samples (Fig. S3). Such a small difference in pore size observed for the last three samples suggests their similar network density, which well supports the observations from rheological studies.

FTIR analysis of the xerogel produced by freeze drying from the SA-3.6-L-3.3 hydrogel was performed, and compared with FTIR spectra of SA and TPDCA (Fig. 4b). The most pronounced differences for the SA-3.6-L-3.3 were found for the FTIR peaks corresponding to the C = O stretching mode in the carboxylic and amidic functionality, which were broadened and shifted to 1725 and 1632 cm^−1^, as compared to those at 1730 and 1636 cm^−1^, respectively, in TPDCA. This can be attributed to the decreasing C = O strength due to the presence of strong hydrogen bonding in the hydrogel. Moreover, the absorption peak of TPDCA at 1530 cm^−1^ belonging to the C = C stretching vibration shifted to 1525 cm^−1^ in the hydrogel. The C = C bond is a part of the 2-pyridone segment from TPDCA molecule, and the decrease of the shift indicates that it is involved in the hydrogen bonding interactions with SA molecules. On the other hand, two absorption peaks at 1597 and 1408 cm^−1^ representing symmetric and asymmetric stretching of C = O group in the carboxylate in SA, respectively, were shifted in the SA-3.6-L-3.3 hydrogel sample to 1600 and 1412 cm^−1^. An increasing stretching of C = O group indicates the decreasing strength of the carboxylic group interacting with a different counterion. In the bare SA, Na^+^ cation with a higher electronegativity provides more electro-deficiency to the C = O group. In the hydrogel sample, interaction of carboxylate with Na^+^ is weakened, and it becomes substituted by TPDCA molecule.

It is evident from the photographs of the TPDCA derived hydrogels (Figs 1 and S3), that the fluorophore provides them with bright light emission. Based on the assumption that TPDCA molecules bearing two carboxyl groups interact with hydroxyl or carboxyl groups of the carbohydrate unit of alginate polysaccharide to form a hydrogel, these interactions could modify HOMO-LUMO transitions of this fluorophore. In the following, we examine UV-Vis absorption, fluorescence excitation and emission spectra, and fluorescence lifetimes of TPDCA fluorophore in ethanol and aqueous solutions, as well as in the alginate/water solution and as a component of the SA hydrogels. In ethanol and water solutions, TPDCA exhibits the n-π* transition band centered at 346 and 347 nm, respectively (Fig. S5). The π-π* transition can also be recognized as a shoulder at ~250 nm. Very similar UV-vis spectra were obtained for the aqueous solution of SA with a low TPDCA concentration (no gelation occurring so far), indicating no influence of their interaction on the HOMO ground state of the fluorophore (Fig. S5). We note that these absorption spectra are in perfect agreement with those reported for the TPDCA fluorophore in water (concentration ~10^−4^ mol L^−1^) earlier^36^. The absorption spectrum of TPDCA in the SA hydrogel could not be measured on our instrumental set-up due to very high concentration of the fluorophore, corresponding to ~10^−1^ mol L^−1^.

**Figure 5:**
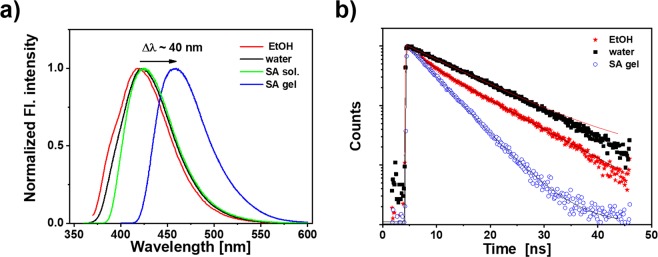
(**a**) Fluorescence spectra (excitation at 350 nm) measured for TPDCA in EtOH, water and 4 wt% alginate/water solution at the concentration 1 × 10^−5^ mol L^−1^ and in the SA-3.6-L-3.3 gel at the concentration 1 × 10^−1^ mol L^−1^; (**b**) Fluorescence decays of TPDCA in EtOH, water, and SA-3.6-L-3.3 hydrogel, measured under 375 nm pulsed laser excitation; solid lines provide the best fits.

Bright fluorescence under 350 nm excitation was observed for TPDCA in ethanol and water, with a rather broad band centered at around 420 nm (exact maxima are at 419 nm and 421 nm in ethanol and water, respectively) (Fig. 5a). As expected for the molecular fluorophores, this emission was independent on the excitation wavelength (Fig. S5). In the SA hydrogel, the fluorescence band was red-shifted by 40 nm, and appeared at 459 nm (Fig. 5a). Such a strong redshift can be due to the high concentration and eventual aggregation of TPDCA molecules interacting with alginate structure. We note that the emission spectrum of TPDCA in the SA/water solution at low concentration (~10^−5^ mol L^−1^) showed emission band at 425 nm, rather similar to 421 nm in pure water (Fig. 5a). Fluorescence excitation spectra of TPDCA were identical to its absorption spectra when detected in EtOH and water, while for TPDCA at high concentration in the alginate hydrogel, much broader and red-shifted band was observed (Fig. S5). This could be a consequence of presence of various light absorbing structures in the ground state (aggregates) at such high TPDCA concentration.

Time-resolved fluorescence measurements provided nearly monoexponential decays for TPDCA in ethanol and water (Fig. 5b), with emission lifetimes equal to 6.9 and 10.1 ns, respectively, well coinciding with previously reported literature values^35^. Quite significant lifetime shortening (down to 4.5 ns) was observed for TPDCA in the SA hydrogel, which can be explained by the fluorophore aggregation at high concentration.

### Gradient hydrogels formed with an aid of divalent ions

**Figure 6:**
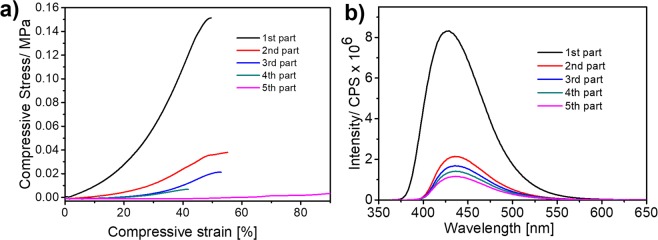
(**a**) Fluorescence intensity of solutions where cut-off cylinders of the gradient gel with Ca^2+^ ions were immersed for 30 min. (**b**) Comparison of the strain measured on 5 cylinders cut-off from the gradient hydrogel with Ca^2+^ ions (counted from the point of immersion).

Hydrogels with gradient properties offer native-like, cellular and biomimetic microenvironments^58,59^, with a number of applications such as biosensing, and soft robotics^60^. We have realized a simple fabrication process for the gradient hydrogels by immersion of a SA-3.6-L-3.3 hydrogel sample in cylindrical set in an aqueous saline solution of divalent cations such as Ca^2+^, Ni^2+^ and Cu^2+^, letting them to diffuse into the hydrogel (Fig. S6). As estimated in a previous study, the primary binding affinity between calcium and alginate was entropically driven, being around 1.33 × 10^6^ mol^−1^ and the second binding affinity was driven by both entropy and enthalpy, being 1.03 × 10^4^mol^−1^ ^61^. Because of the interactions of alginate with calcium ions and other molecules are predominately competitive in nature, we expect that these divalent ions have much stronger affinity to alginate, and may replace the hydrogen bonded TPDCA and sodium ions to form a dynamic egg-box-like structure^23^. Such replacement of the TPDCA fluorophore with e.g. Ca^2+^ ions would result in its gradual release from the hydrogel, which could be directly observed on the set of samples which were pushed out from a syringe mold, cut into five 2 mm long cylinders, and immersed to 0.9 wt% NaCl saline solution. An intense blue fluorescence from the firstly cut cylinder has been observed, while the fluorescence intensity gradually decreased upon increasing the distance from the immersion point (Fig. 6a). This observation clearly indicates a dynamic replacement of the TPDCA crosslinker by divalent Ca^2+^ ions with a higher affinity to alginate^62^. This is also supported by fluorescence measurement on TPDCA released into the aqueous saline solution, where intensity gradually decreased from the first fraction after immersion to the last one with a comparatively weak fluorescence intensity. The same cut-off cylinders were subjected to the compression test (Fig. 6b), showing the highest compression stress of 160 kPa for the 1^st^ cylinder from the immersion point, which decreased to 40 kPa for the 3^rd^ cylinder, and to only 4 kPa indicating formation of a soft material for the last 5^th^ cylinder.

The formation of gradient hydrogels is not limited to Ca^2+^ ions but is general for other divalent ions such as Ni^2+^ and Cu^2+^ ions; it can be observed both visually, through the change of color as these ions absorb in the visible part of the spectrum and through gradually weakening fluorescence of TPDCA, as demonstrated in Fig. S6a. In the case of Cu^2+^ and Ni^2+^ ions, the highest compression stress for the first cut-off cylinder reached 118 kPa and 115 kPa, respectively, and experienced gradual decrease similarly as for the Ca^2+^ based gradient hydrogel, for the subsequent cylinders (Fig. S7). The decrease was the lowest in the case of Cu^2+^ ions, which is in a good agreement with reported affinity of multivalent metal ions to alginate^63^.

## Conclusions

We introduced a novel approach for the fabrication of fluorescent alginate hydrogels without using multivalent ions as gelation agents, but crosslinked with a molecular fluorophore TPDCA, instead. This molecular fluorophore is also formed as an emissive component in the hydrothermal synthesis of carbon dots, which are prepared from natural sources citric acid and L-cysteine at rather low temperatures. TPDCA has been identified as the major segment responsible for gelation of alginate, which happens after some induction period through the hydrogen bonding of the carboxylic functionality of this molecule with hydroxyl and carboxylate groups of the alginate to form a dynamic hydrogel network structure. The resulting TPDCA derived hydrogels combine an intense blue fluorescence of this molecular fluorophore with the excellent self-healing properties and viscoelastic characteristics tunable by the amount of alginate. Furthermore, gradient hydrogels with modulated mechanical and optical properties and controlled release can easily be prepared by immersion of the TPDCA derived hydrogels into divalent metal ion solutions. Such a simple fabrication process of hydrogels shows potential in modulation of their mechanical and releasing properties which can be useful for soft robotics, food and cosmetic industry.

## Experimental

### Materials

Citric acid (CA, ACS reagent 99.5%), CaCl_2_.2H_2_O (Bioreagent, ≥99%), CuCl_2_ (98%), NiCl_2_.6H_2_O (99.999% trace metals basis), graphene oxide (GO, 4 mg/mL, dispersion in H_2_O) and L-cysteine (≥97%) were purchased from Sigma-Aldrich and were used as received. Sodium alginate (SA, Sigma-Aldrich, CAS 9005-38-3) with G/M monomer unit ratio of 1.1 was used as received. Reduced graphene oxide (rGO) was prepared by reduction of GO using an access of ascorbic acid in water solution^64^. Absolute ethanol (99.5%, Centralchem, Slovakia) was used as received. Deionized water (DI water) was obtained from a milliQ Millipore setup and used in all the experiments.

### Synthesis of TPDCA

5-oxo-2,3-dihydro-5H-[1,3]-thiazolo[3,2-a]pyridine-3,7-dicarboxylic acid (TPDCA) was synthesized according to ref.^42^ and its structure was confirmed by ^1^H and ^13^C NMR spectra (Figs SI 1 and 2, respectively).

^1^H NMR (d_6_-DMSO) δ (ppm): 3.68 and 3.98 (2 H, d, -CH_2_-); 5.53 (1 H, s, CH-); 6.61(2 H, d, = CH-), 6.66 (2 H, d, = CH-), 13.5 (2 H, broad s, COO-H).

^13^C NMR (d_6_-DMSO) δ (ppm): 31.6 (C-7); 62.6 (C-6); 97.9 (C-2); 115.0 (C-4); 142.7 (C-3 arom.); 150.0 (C-5 arom.); 160.6 (C-1 keto); 165.6 (C-8 carbox.); 169.2 (C-9 carbox.).

### Synthesis of CDs

2.87 g of CA and 1.81 g of L-cysteine were dissolved in 30 mL DI water, loaded into a 150 mL Teflon lined stainless steel autoclave and heated at 120, 150 and 200 °C for 4 h. After natural cooling, the reaction mixture was dialyzed in a dialysis tube (molecular weight cut-off 3500) by several water exchanges, and dried.

### Hydrogel preparation

In a typical gelation process, appropriate amount of SA was dissolved in 800 µL of DI water, and heated at 60 °C for 2–3 h in a clean glass vial. Similarly, 36 mg each of either TPDCA, CDs, GO or rGO were dissolved or dispersed, respectively, in 300 µL of DI water at elevated temperature. Once the contents in both vials were cooled to room temperature, both solutions were mixed together using Vortex mixer, kept in the ultrasonic bath for 15 min, and left overnight. Gelating abilities of different applied materials and conditions were estimated by inversion of vials and steel ball techniques; they are summarized in Table 1. The sample codes for hydrogels which were produced in this study include the prefix with sodium alginate (SA) followed by number providing its relative amount in wt%; followed by another prefix for the gelation reagent used (TPDCA = L, CD, GO or rGO) and its respective relative amount in wt%.

### Preparation of gradient hydrogels under employment of divalent ions

The SA-3.6-L-3.3 hydrogel was loaded into a syringe cylinder (1 ml syringe, BBraun, with a cut-off top) as a molding form before its gelation, and the top of the cylinder was sealed with parafilm. After completion of the gelation process, the sealing was removed and the cylinder was dipped vertically into multivalent metal ion aqueous solutions of either CaCl_2_ (30 mg/mL), NiCl_2_ (60 mg/mL), or CuCl_2_ (60 mg/mL). Formation of the metal ion gradient was observed along the gel cylinders, visualized by the color of the respective metal ions or solid white (in the case of Ca^2+^). After completion of diffusion of divalent ions, the resulting gel was pushed out from the mold into a physiological solution (0.9 wt % NaCl). The gel cylinder was cut into five 2 mm equal sized cylinders, which were immersed in individual physiological solutions for 30 min. Compression and fluorescence measurements were subsequently performed on the cylinders and these solutions, respectively.

### Spectral measurements

^1^H NMR and ^13^C NMR spectra were recorded at 298 K using a JNM-ECZ600R instrument (Jeol). The chemical shifts were presented in ppm downfield from an internal standard TMS (0.00 ppm), using the solvent as a reference. The working frequencies were 600 MHz for ^1^H and 150 MHz for ^13^C NMR; the abbreviations used for denotion of spectra are as follows: s = singlet, d = doublet. Fourier transform infra-red (FTIR) spectra were obtained on a Frontier FT IR spectrometer (Perkin Elmer). Absorption spectra were recorded on a UV 1650PC spectrophotometer (Shimadzu, Japan). Fluorescence spectra were recorded on a RF-5301PC spectrofluorophotometer (Shimadzu, Japan), and on a FluoroMax-4 spectrofluorometer (Horiba Yvon-Jobin, Japan), using 1 × 1 cm quartz cuvette. All fluorescence spectra were collected by excitation at the maximum of the longest wavelength absorption band. For the samples with high concentration of fluorescent fluorophore, the cuvettes were placed in the solid sample holder in the front-face arrangement, and the emission from the sample was detected under 40° angle from the surface between the solution and glass, so that to decrease the emission self-quenching. Fluorescence decay spectra were collected on a time-correlated single-photon counting (TCSPC) setup, which is analogous to the one escribed in detail previously^65^ (all components were obtained from Becker&Hickl GmbH, Berlin, Germany). A brief description of the measurement procedure is as follows: the sample was excited by a 375 nm picosecond diode laser with an output power of ~1 mW, pulse widths of app. 50 ps and a frequency of 20 MHz. The fluorescence was spectrally separated from the laser excitation using a 395 nm dichroic filter and a 397 nm long-pass filter. A polarizer in a magic-angle orientation was fitted in front of the detection system to prevent distortions of the decay kinetics due to depolarization effects. The emission was measured by a 16-channel multi-anode photomultiplier array attached to the 160 mm spectrograph (PML-SPEC). The PML detector was operated in the photon-counting regime.

### Rheological studies

The rheological properties of the gels were measured on a rotational rheometer MCR-502 (Anton Paar, Austria) with parallel plate geometry of 10 mm in diameter and 1000 μm in thickness, at 20 °C controlled with a Peltier setup. Frequency dependences of the storage (*G*′) and elastic (*G*′′) moduli from 0.1 to 10 Hz were measured in the linear viscoelastic region, which was established at 1.58% strain (γ) value. The recovery tests were performed according to a similar procedure with the same setup at strain deformations from 0.01 to 1000% at a frequency of 1 Hz followed by measurements of the time dependence of the G′ at linear conditions (1.58% strain deformation and 1 Hz) to monitor the recovery of the storage modulus (G′) and finally the whole mechanical properties. All measurements were performed at 25 °C. To avoid the dehydration of the samples, the solvent trap from Anton Paar was used. The kinetics of cross-linking reactions was studied at similar linear conditions as mentioned above (1.58% strain deformation and 1 Hz) for up to 10 h at 25 °C.

### Microscopy

Morphology of the samples was studied by scanning electron microscopy (SEM) using JSM 6610 microscope (Jeol) at accelerating voltage of 15 kV. The samples were sputtered with a thin layer of gold. AzTec software was used for collecting and processing of the data.

### Mechanical tests

The metal ion diffused gels were subjected to mechanical strain, and the break behavior was studied using dynamic mechanical analyzer (DMA, TA-RSA-G2) in the compression mode and axial direction. Equal-sized test samples were cut and the sample cylinders were placed on the DMA stage for analysis, in a way that their flat surface lied in the horizontal direction while the tube-like cylindrical surface was settled in the vertical direction. This positioning ensured a homogenous distribution of the applied strain throughout the gel material.

## Supplementary information

Supplementary information
