# Supplementary material for: Identification of Molecular Fluorophore as a Component of Carbon Dots able to Induce Gelation in a Fluorescent Multivalent-Metal-Ion-Free Alginate Hydrogel
Source: Sci Rep. 2019 Oct 21;9:15080. doi: 10.1038/s41598-019-51512-2 (PMC6803645; doi:10.1038/s41598-019-51512-2)
Supplement: Supplementary file 1 — Supplementary information [file 41598_2019_51512_MOESM1_ESM.docx]

**Supporting information**

**Identification of Molecular Fluorophore as a Component of Carbon Dots able to Induce Gelation in a Fluorescent Multivalent-Metal-Ion-Free Alginate Hydrogel**

Peter Kasak,^*,1^ Martin Danko,^1,2^ Sifani Zavahir,^1^ Miroslav Mrlik,^3^ Yuan Xiong,^4^ Ammar Bin Yousaf,^1^ Wing-Fu Lai,^5^ Igor Krupa,^1^ Jan Tkac,^6^ and Andrey L. Rogach^*,4^

^1^Center for Advanced Materials, Qatar University, P.O. Box 2713 Doha, Qatar co

^2^Polymer Institute, Slovak Academy of Sciences, Dúbravská cesta 9, 845 41 Bratislava, Slovak Republic

^3^Centre of Polymer Systems, University Institute, Tomas Bata University in Zlín, Trida T. Bati 5678, 760 01, Zlín, Czech Republic

^4^Department of Materials Science and Engineering, and Center for Functional Photonics (CFP), City University of Hong Kong, 83 Tat Chee Avenue, Kowloon, Hong Kong S.A.R.

^5^School of Pharmaceutical Sciences, Health Science Center, Shenzhen University, Shenzhen, China

^6^Department of Glycobiotechnology, Institute of Chemistry, Slovak Academy of Sciences, Dúbravská cesta 9, 845 38 Bratislava, Slovak Republic

Correspondence and requests for materials should be addressed to P.K. (email: [peter.kasak@qu.edu.qa](mailto:peter.kasak@qu.edu.qa)) or A.L.R. (email: [andrey.rogach@cityu.edu.hk](mailto:andrey.rogach@cityu.edu.hk) )

Content:

1. Figure SI1: a) ^1^H and b) ^13^C NMR spectra of 5-oxo-2,3-dihydro-5H-[1,3]-thiazolo[3,2-a]pyridine-3,7-dicarboxylic acid (TPDCA).
2. Figure SI2: Images of samples a) SA-x-CQD120, b) SA-x-CQD150, c) SA-x-CQD200 and d) SA-x-GO where x is amount of SA (x = 1.8, 3.6, 5.4, 7.2 wt%) at normal day light (left) and after UV light exposure (365 nm) (right).
3. Figure SI3. SEM images for freeze dried sample SA-3.6-L-3.3 and SA-3.6-L-7.2.
4. Figure SI4: Absorption spectra of TPDCA in EtOH, water and Alginate solution at concentration 1×10^-5^ mol L^-1^.
5. Figure SI5: Emission spectra of TPDCA in EtOH and water solution at concentration 1×10^-5^ mol L^-1^ performed at different excitation.
6. Figure SI6: Fluorescence emission spectra of SA-5.4-CQD-150 at 1×10^-1^ mol L^-1^ performed at different excitation.
7. Figure SI7: Fluorescence excitation (em. 420 nm) spectra of TPDCA in EtOH and water solution at concentration 1×10^-5^ mol L^-1^ and in SA-3.6-L-3.3 gel at 1×10^-1^ mol L^-1^.
8. Figure SI8: Fluorescence excitation spectra of SA-5.4-CQD-150 at 1×10^-1^ mol L^-1^ performed at different emissions.
9. Figure SI9: Fluorescence emission spectra of TPDCA in 3.6 wt% alginate/water solution at concentration 1×10^-1^ mol L^-1^ performed at 350 nm excitation before and after gelation.
10. Figure SI10. Image of gradient hydrogel procedure.

*
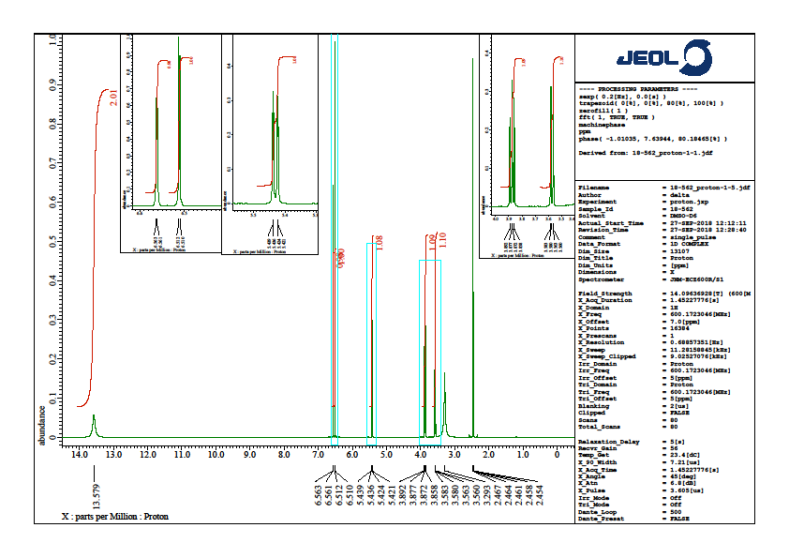
*

**b)**

**a)**


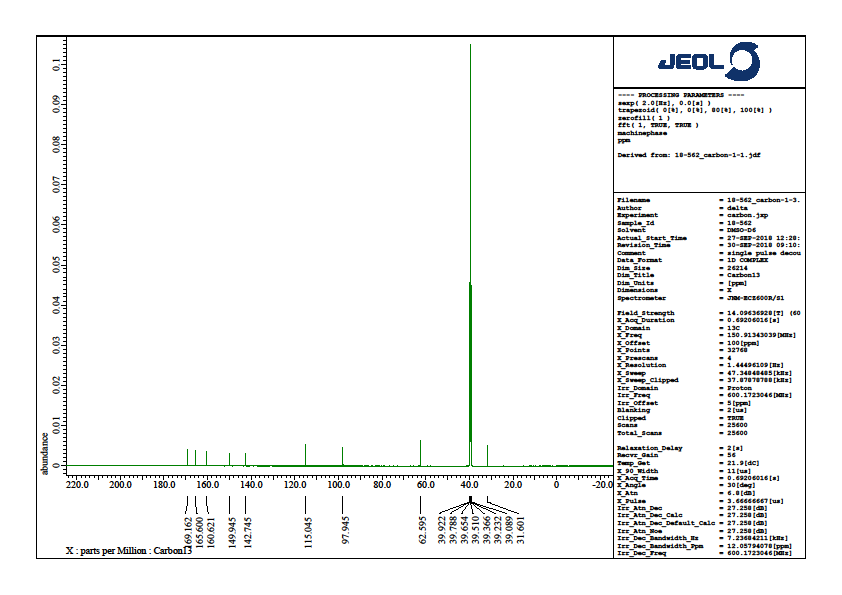


FigureSI1. a) ^1^H and b) ^13^C NMR spectra of 5-oxo-2,3-dihydro-5H-[1,3]-thiazolo[3,2-a]pyridine-3,7-dicarboxylic acid (TPDCA).


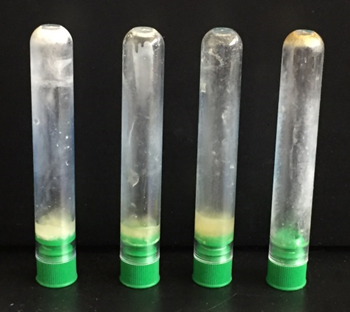

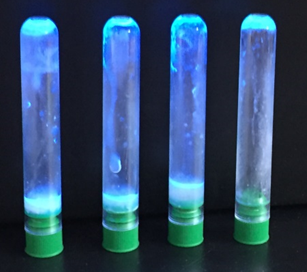


**b)**

**a)**


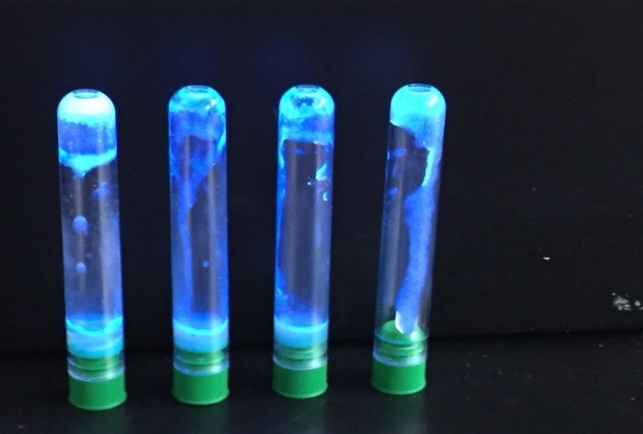

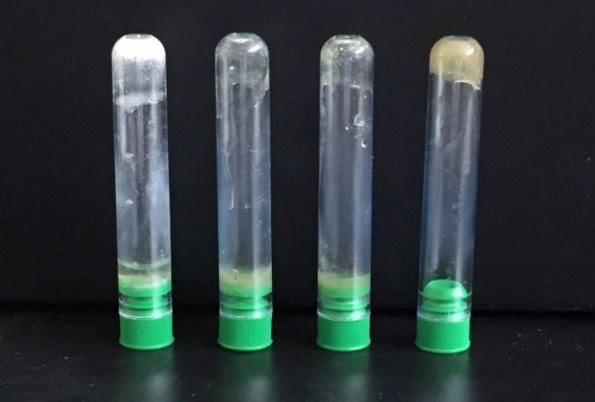


*
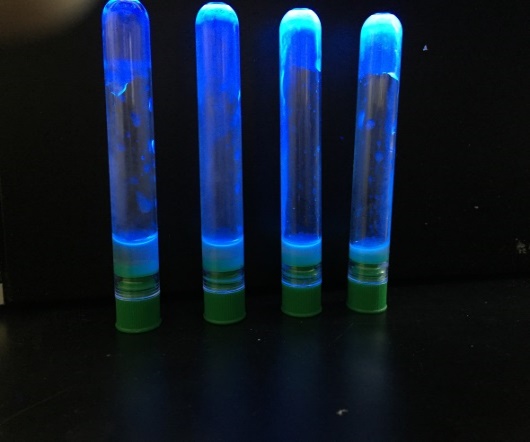
*

**c)**

**d)**


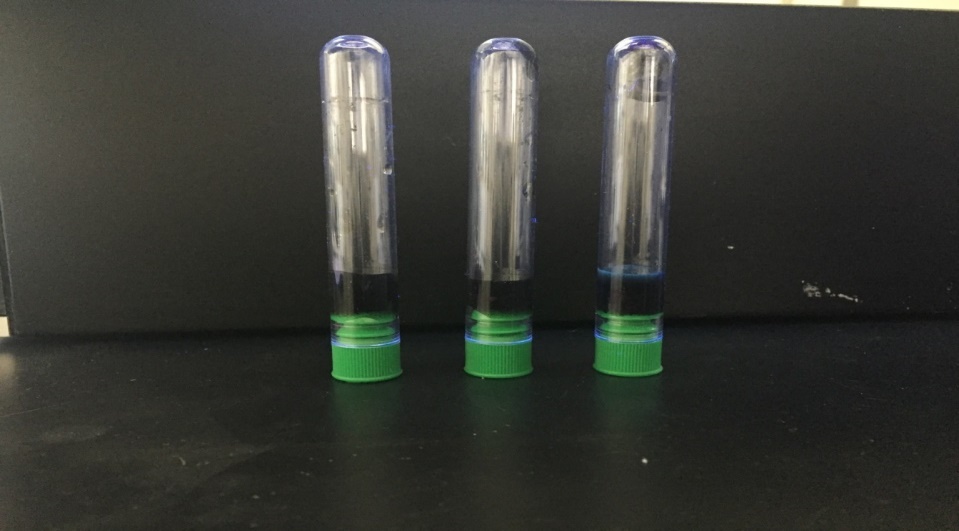

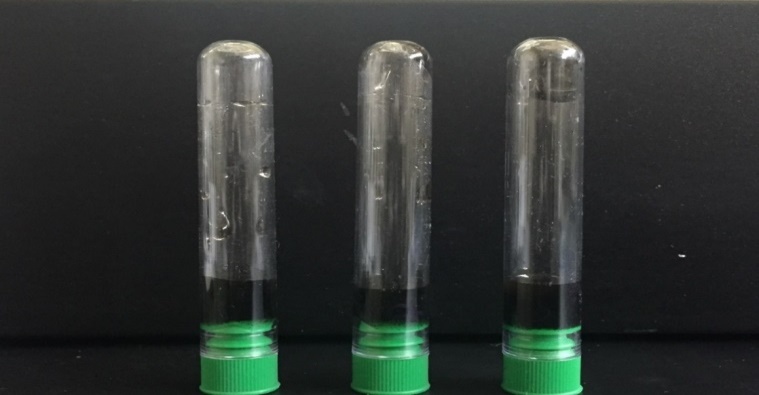


Figure SI2: Images of samples a) SA-x-CQD120, b) SA-x-CQD150, c) SA-x-CQD200 and d) SA-x-GO where x is amount of SA (x = 1.8, 3.6, 5.4, 7.2 wt%) at normal day light (left) and after UV light exposure (365 nm) (right).


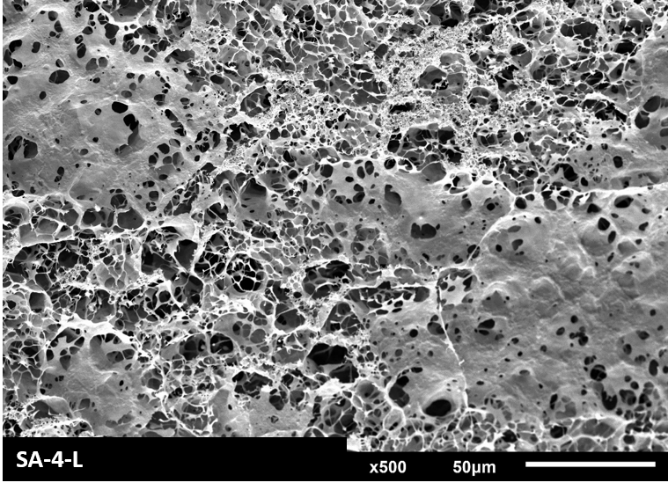

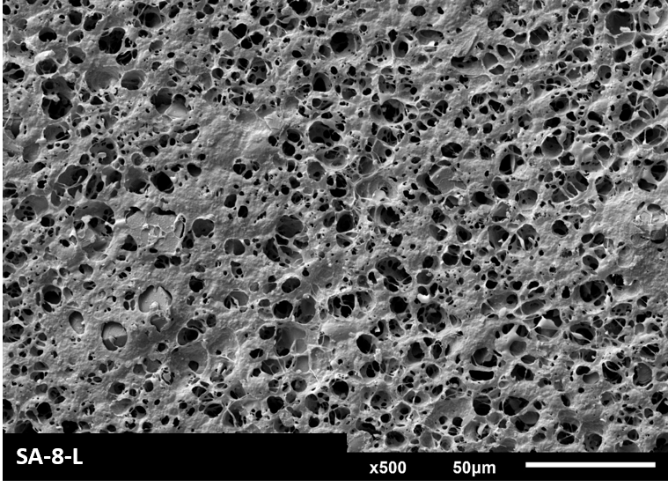


Figure SI3: SEM images for freeze dried sample SA-3.6-L-3.3 and SA-3.6-L-7.2.

Figure SI4: Absorption spectra of TPDCA in EtOH, water and Alginate solution at concentration 1×10^-5^ mol L^-1^.

Figure SI5: Fluorescence emission spectra of TPDCA in EtOH and water solution at concentration 1×10^-5^ mol L^-1^ performed at different excitation.

Figure SI6: Fluorescence emission spectra of SA-5.4-CQD-150 at 1×10^-1^ mol L^-1^ performed at different excitation.

Figure SI7: Fluorescence excitation (em. 420 nm) spectra of TPDCA in EtOH and water solution at concentration 1×10^-5^ mol L^-1^ and in SA-3.6-L-3.3 gel at 1×10^-1^ mol L^-1^.

Figure SI8: Fluorescence excitation spectra of SA-5.4-CQD-150 at 1×10^-1^ mol L^-1^ performed at different emissions.

Figure SI9: Fluorescence emission spectra of TPDCA in 3.6wt% alginate/water solution at concentration 1×10^-1^ mol L^-1^ performed at 350 nm excitation before and after gelation.


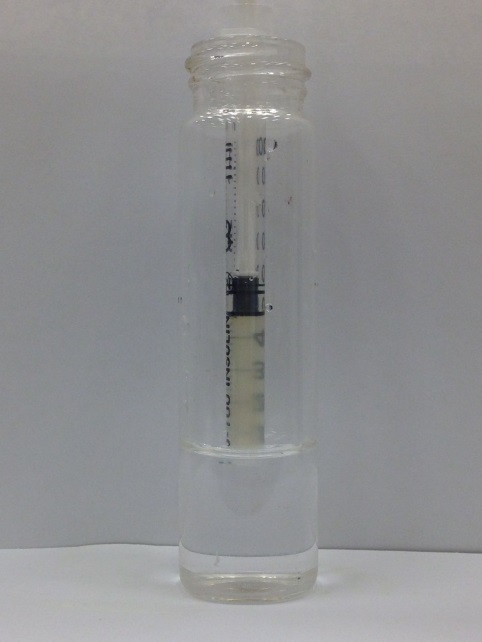


Figure SI10. Image of gradient hydrogel procedure.
